# An Evaluation of Statistical Approaches to Rare Variant Analysis in Genetic Association Studies

**DOI:** 10.1002/gepi.20450

**Published:** 2009-10-06

**Authors:** Andrew P Morris, Eleftheria Zeggini

**Affiliations:** 1 Wellcome Trust Centre for Human Genetics, University of OxfordOxford, United Kingdom; Wellcome Trust Sanger InstituteHinxton, Cambridge, United Kingdom

**Keywords:** rare variant association, re-sequencing data, genome-wide association data

## Abstract

Genome-wide association (GWA) studies have proved to be extremely successful in identifying novel common polymorphisms contributing effects to the genetic component underlying complex traits. Nevertheless, one source of, as yet, undiscovered genetic determinants of complex traits are those mediated through the effects of rare variants. With the increasing availability of large-scale re-sequencing data for rare variant discovery, we have developed a novel statistical method for the detection of complex trait associations with these loci, based on searching for accumulations of minor alleles within the same functional unit. We have undertaken simulations to evaluate strategies for the identification of rare variant associations in population-based genetic studies when data are available from re-sequencing discovery efforts or from commercially available GWA chips. Our results demonstrate that methods based on accumulations of rare variants discovered through re-sequencing offer substantially greater power than conventional analysis of GWA data, and thus provide an exciting opportunity for future discovery of genetic determinants of complex traits. *Genet. Epidemiol*. 34: 188–193, 2010. © 2009 Wiley-Liss, Inc.

## INTRODUCTION

Recent advances in whole genome genotyping technologies, the availability of large, well-defined population-based disease cohorts, and a better understanding of common human sequence variation, coupled with the development of appropriate quality control and analysis pipelines, have led to the identification of many novel common genetic determinants of complex traits [The Wellcome Trust Case Control Consortium, 2007; Zeggini et al., 2008; Barrett et al., 2008; Raychaudhuri et al., 2008; Cooper et al., 2008; Willer et al., 2009; Aulchenko et al., 2009; Prokopenko et al., 2009]. Nevertheless, despite these successes, much of the genetic component of these traits remains unaccounted for. Although there may be many undiscovered common polymorphisms associated with complex traits, it seems unlikely that the “common-disease common-variant” hypothesis is all encompassing. One unexplored paradigm which may contribute to this unexplained genetic component is a model of multiple rare causal variants, defined here to have a minor allele frequency (MAF) of less than 1%, each of modest effect, but residing within the same functional unit, for example, a gene. Joint analysis of rare variants within a gene, searching for accumulations of minor alleles within the same individual, may thus provide signals of association with complex phenotypes that could not have been identified through traditional association analysis of single nucleotide polymorphisms (SNPs), typically defined to have MAF of at least 1%. For example, minor alleles at multiple rare variants in *ABCA1, APOA1* and *LCAT* have been demonstrated to contribute collectively to low plasma levels of high-density lipoprotein cholesterol [Cohen et al., 2004].

Currently, most studies of rare variants utilise data from commercially available GWA chips, which are far from ideal since they are designed for capturing common human genetic variation. However, the availability of data more appropriate for rare variant association analysis is just around the corner, with whole genome re-sequencing efforts, such as the 1,000 Genomes project (http://www.1000 genomes.org) soon reaching completion. Furthermore, large-scale deep re-sequencing technologies are becoming increasingly efficient and cost effective, and thus may soon be realistic for rare variant discovery in specific genes in large disease or population-based cohorts. We have developed a novel test of association with rare variants discovered through such re-sequencing efforts, based on the accumulation of minor alleles within the same functional unit, for example a gene-coding region extended up and downstream to incorporate additional functional elements and the regulatory region. We have then undertaken a simulation study to focus on two distinct, but timely, scenarios with the aim of addressing specific, as yet unanswered, methodological questions in each. First, when deep re-sequencing data are available to discover rare variants, do methods based on accumulations of minor alleles within the same functional unit offer greater power to detect association with complex traits than traditional analysis of SNPs on GWA chips? Second, when only GWA chip data are available, what is the most powerful strategy for identifying rare variant associations with complex traits?

## METHODS

We consider two specific tests of quantitative trait association with accumulations of minor alleles across rare variants within the same functional unit. In the first of these tests, the phenotype is modelled in a linear regression framework as a function of the *proportion* of rare variants at which an individual carries a minor allele. In the second, the phenotype is modelled in the same regression framework, but this time as a function of the presence/absence of a minor allele at *any* rare variant within an individual. This collapsing approach has been previously proposed in the context of a binary trait [Li and Leal, 2008], and has been demonstrated to be powerful for detecting association with rare variants discovered through re-sequencing.

Consider a sample of unrelated individuals, phenotyped for a normally distributed trait, and typed for rare variants in a gene or small genomic region. Let *n_i_* denote the number of rare variants for which the *i*th individual has been successfully genotyped, and let *r_i_* denote the number of these variants at which they carry at least one copy of the minor allele. We can model the phenotype, *y_i_*, of the *i*th individual in a linear regression framework, given by *y_i_* = E[*y_i_*]+ɛ_*i*_, where ɛ_*i*_,σ_*E*_), and


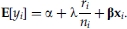


In this expression, *x_i_* denotes a vector of covariate measurements for the *i*th individual, with corresponding regression coefficients β. The parameter λ is the expected increase in the phenotype for an individual carrying a full complement of minor alleles at rare variants compared to an individual carrying none. As an alternative, we can model the expected phenotype of the *i*th individual as


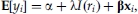


where *I(r_i_)* is an indicator variable taking the value 1 if *r_i_*>0, and 0 otherwise, in other words, the presence of at least one minor allele at any rare variant. Here, the parameter λ is the expected increase in the phenotype for an individual carrying at least one minor allele at any rare variant compared an individual carrying none.

For either model, the likelihood contribution of the *i*th individual is given by


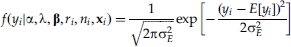


We thus construct likelihood ratio tests of association of an accumulation of rare variants with disease by comparing the maximised likelihoods of two models via analysis of deviance: (i) the null model where λ = 0; and (ii) the alternative model for which λ is unconstrained. The contribution of the *i*th individual to the likelihood, *f(y_i_* | α,λ,β,*r_i_*,*n_i_*,*x_i_*), is weighted by *n_i_* to allow for differential call rates between samples. We denote the likelihood ratio test based on the proportion of rare variants at which an individual carries minor alleles by RVT1, and that based on the presence/absence of at least one minor allele at any rare variant by RVT2. Both RVT1 and RVT2 can be generalised to tests of association with a binary trait within a logistic regression-modelling framework.

## SIMULATION STUDY

In order to evaluate the relative merits of different analytical approaches to identify rare variant associations with a quantitative trait, we have performed simulations using simple models of population genetics to generate high-density haplotype data in a 50-kb genomic region used to represent a functional unit of interest. We considered a range of models for association of the trait with multiple causal variants in the same region, under two different assumptions: (i) the mean trait value is determined by the presence or absence of a minor allele at *any* causal variant; and (ii) the mean trait value determined by the *proportion* of causal variants at which a minor allele is present. Trait association models were then parame-terised in terms of: (i) the maximum MAF of any individual causal variant; (ii) the total MAF of all causal variants; and (iii) their joint contribution to the phenotypic variance. Full details of the simulation process are described in the Appendix.

We began by simulating a population of 40,000 haplotypes, and selected causal variants according to our chosen model of association. We selected 10,000 haplotypes, paired together at random to form 5,000 individuals in our “analysis cohort”, and generated their phenotypes according to their genotypes at the causal variants. We then selected a further 2,000 haplotypes in our “discovery panel”, used here to represent the deep re-sequencing data we expect from the 1,000 Genomes project. Over all simulations, the mean number of rare variants with at least two copies of the minor allele in the discovery panel was 52.2. We assumed that each of these rare variants was taken forward for genotyping in the analysis cohort, and tested for association using both RVT1 and RVT2.

Our next step was to select variants in the 50 kb region to have similar properties to the Affymetrix Human SNP Array 6.0 in terms of mean density and MAF profile in the population of 40,000 haplotypes. All SNPs on the GWA chip were analysed independently, using conventional trend tests of association, with Bonferroni correction for multiple testing, and jointly, using standard haplotype-based techniques. Over all simulations, the mean number of GWA chip SNPs in the region was 14.8, while the mean number of SNP haplotypes with population frequency greater than 1% was 18.0. We attempted to apply RVT1 and RVT2 to the GWA chip data, but the mean number of rare variants in the region was just 0.2, and provided minimal power to detect accumulations of minor alleles at these loci. As a result, we extended our analysis to include “low-frequency” SNPs (1%<MAF<5%), which are less scarce on the GWA chip (mean 1.6 variants in the region).

**Fig. 1:**
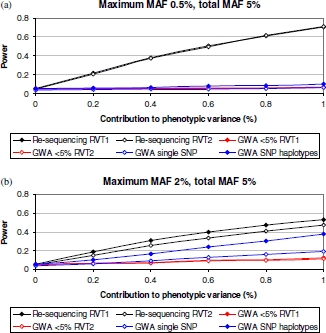
Power of six tests of rare variant association with a quantitative trait as a function of the percentage of phenotypic variation explained by causal variants in a 50 kb region, assuming the trait mean is determined by the presence or absence of minor alleles at any of the causal variants. Results for two models are presented, both assuming a total MAF of 5% for all causal variants in the region: (a) the maximum MAF of any individual causal variant is 0.5% and (b) the maximum MAF of any individual causal variant is 2%. Power is estimated at a 5% significance level over 10,000 replicates of data. Re-sequencing RVT1: test of phenotype association with the proportion of rare variants, discovered through re-sequencing, at which individuals carry minor alleles. Re-sequencing RVT2: test of phenotype association with the presence/absence of minor alleles in individuals at any rare variant discovered through re-sequencing. GWA <5% RVT1: test of phenotype association with the proportion of low-frequency variants on the GWA chip at which individuals carry minor alleles. GWA <5% RVT2: test of phenotype association with the presence/absence of minor alleles at any low-frequency variant on the GWA chip. GWA single SNP: standard trend test of quantitative trait association with each SNP on the GWA chip, with Bonferroni correction for multiple testing. GWA SNP haplotypes: haplotype trend test of association with the quantitative trait across all SNPs on the GWA chip.

Figure 1 shows the power of each of the tests of association as a function of the percentage of phenotypic variation explained by causal variants in the 50 kb region, assuming the trait mean is determined by the presence or absence of minor alleles at any of the causal variants. Results for two models are presented here, each assuming a total MAF of 5% for all causal variants in the region: (a) the maximum MAF of any individual causal variant is 0.5%; and (b) the maximum MAF of any individual causal variant is 2%. Model (b) incorporates fewer and, on average, more common causal variants than does (a), and thus represents a lower degree of allelic heterogeneity. Supplementary Figures 1 and 2 present power for a wider range of association models encompassing intermediate levels of allelic heterogeneity, where trait means are determined by the presence or absence of minor alleles at any causal variant, and by the proportion of causal variants at which a minor allele is present, respectively.

Our results highlight a number of general conclusions. First, when rare variants are discovered through re-sequencing, RVT1, based on the proportion of rare variants at which an individual carries minor alleles, is always at least as powerful as RVT2, based on the presence/absence of minor alleles. The difference in power between the two tests is most noticeable when the trait mean is determined by the proportion of causal variants at which a minor allele is present, which is not surprising, since this model is assumed by RVT1 (Supplementary Fig. 2). However, even when the trait mean is determined by the presence or absence of a minor allele at any causal variant, RVT1 is generally more powerful than RVT2. This would suggest that RVT2 is less robust to the presence of minor alleles at non-causal rare variants than is RVT1.

Next, there is a clear gain in power for tests based on rare variants identified through re-sequencing over analyses of SNPs or low-frequency variants present on the GWA chip. The greatest gains are observed in the presence of substantial allelic heterogeneity (Fig. 1a), where rare causal loci are less likely to be captured by SNPs as a result of linkage disequilibrium [The International HapMap Consortium, 2005; Zeggini et al., 2005]. However, the differences in power between the tests are less noticeable when there is less allelic heterogeneity (Fig. 1b). Our results also confirm previous findings that haplotype-based analyses of SNPs have greater power to detect rare variant associations than single-locus tests, unless there is substantial allelic heterogeneity [Morris and Kaplan, 2002]. Finally, low-frequency variants (MAF<5%) on GWA chips are too scarce to detect accumulations of minor alleles, and thus RVT1 and RVT2 have minimal power to identify rare variant associations with this type of data.

## DISCUSSION

Our simulations clearly indicate that tests based on the accumulation of minor alleles at rare variants identified through re-sequencing are always more powerful than conventional tests applied to SNPs present on GWA chips, particularly in the presence of substantial allelic heterogeneity. We have assumed a discovery panel of 1,000 individuals from the same population from which the analysis cohort has been ascertained which may not always be the case. With the expense of re-sequencing efforts, focussed studies of samples from the analysis cohort are likely to be much smaller, and thus less powerful for rare variant discovery, although pooling may provide a more efficient initial screening step. Publicly available re-sequencing panels, such as those that will be released through the 1,000 Genomes project may not be matched for ancestry with the analysis cohort. These panels will miss rare variants specific to the population from which the analysis cohort has been ascertained, and may lead to genotyping of variants which are, in fact, monomorphic.

Our simulations also assume that all rare variants identified through re-sequencing of the discovery samples will subsequently be genotyped in the analysis cohort. However, genotyping these rare variants on a genome-wide scale will be a considerably more expensive endeavour than utilising GWA platforms. This approach may currently be financially infeasible with the large samples required to detect the modest genetic effects we expect for complex traits, particularly for rare variant associations. One possible approach to reduce genotyping costs is to focus on potentially functional rare variants (for example those leading to non-synonymous changes or located within exons and regulatory regions). However, at present, there is no unbiased evidence to suggest that causal variants are more likely to aggregate in such regions and current annotation of the genome is incomplete, making identification of potentially functional loci difficult.

Experience with current genotyping technologies would suggest that rare variants are more difficult to type than SNPs, and thus stringent quality control procedures are required to avoid increased false-positive error rates as a result of genotype misspecification. To increase power, an obvious step would be to combine results of rare variant studies through meta-analysis. Although each study may genotype different loci, we can combine results on the level of the functional unit. This may reduce, or even eliminate, the need for imputation, which may be potentially prone to bias because the spectrum of rare variants is more diverse than that of SNPs, even between populations sharing relatively recent common ancestry, and therefore more difficult to predict [Anderson et al., 2008]. This highlights the need for replication in large samples from closely related populations to confirm rare variant association signals.

The field of complex trait genetics is moving rapidly towards an understanding that deep re-sequencing technologies will provide the necessary data to unearth novel-associated loci. It is anticipated that researchers will soon be faced with the challenge of selecting the appropriate analytical strategy for these data sets, which will be of unprecedented scale and depth. In this study, we have developed and evaluated targeted rare variant analysis methods and have provided insights into their relative merits. The methodology we have developed here for detecting rare variant associations is extremely simplistic, and as technologies probing human genome sequence variation move rapidly forward, the development and testing of analytical strategies that maximise output from these investments will continue to be of critical importance.

## APPENDIX

We have performed simulations to evaluate the relative merits of different analytical approaches to identify rare variant associations with a quantitative trait: (i) tests of association of rare variants (MAF <1%) discovered through re-sequencing based on accumulations of minor alleles in the extremes of the phenotype distribution; (ii) the same tests applied to low-frequency variants (MAF <5%) on GWA chips and (iii) conventional single-locus and haplotype-based tests of association with SNPs (MAF >1%) on GWA chips. We considered a range of models for association of the trait with multiple causal variants in the same region, under two different assumptions: (i) the mean trait value is determined by the presence or absence of a minor allele at *any* causal variant and (ii) the mean trait value determined by the *proportion* of causal variants at which a minor allele is present. Trait association models were then parameterised in terms of: (i) the maximum MAF, δ, of any individual causal variant; (ii) the total MAF, *Q,* of all causal variants and (iii) their joint contribution to the phenotypic variance, expressed as 100λ%. For each model, we generated 10,000 replicates of data as follows:

1. Generate an ancestral recombination graph [Griffiths and Marjoram, 1997] for a population of 40,000 haplotypes from a realisation of the coalescent process with recombination, obtained using the MS software [Hudson, 2002]. We assumed a mutation rate of 10^−8^ per base (in each generation) and a recombination rate of 1 cM per Mb, for an effective population size of 10,000 individuals, corresponding to scaled recombination and mutation rates of ρ = θ = 20 across the 50 kb region [Nordborg, 2001].
2. Calculate the MAF at each variant across the popula tion, denoted *q_j_* for the *j*th locus. Select a random subset of variants as causal, each with MAF *q_j_*<δ, and with total MAF of approximately *Q*.
3. Select a random sample of 10,000 chromosomes from the population, paired together at random to form an “analysis cohort” of 5,000 individuals to be genotyped for association testing. Determine the number of minor alleles across all causal variants carried by the *i*th individual, denoted *m_i_*. Under the assumption that the trait mean is determined by the presence or absence of minor alleles at any causal variant, we simulate the phenotype, *y_i_*, of the *i*th individual from a N(*I(m_i_* > 0),σ) distribution. Conversely, under the assumption that the trait mean is determined by the proportion of causal variants at which a minor allele is present, we simulate the phenotype, *y_i_*, from a N(*m_i_*,σ) distribution. In both scenarios, the standard deviation, σ, is determined by the spectrum of causal variants and their joint contribution, λ to the phenotypic variance.
4. Select a random sample of 2,000 chromosomes from the population, representing 1,000 individuals in the “dis covery panel”. Identify all variants that have at least two occurrences of the minor allele, but with MAF of less than 1%, in the discovery panel. Record the genotypes of each individual in the analysis cohort at all rare variants identified in the discovery panel. Apply the two proposed tests of quantitative phenotype association with accumulations of minor alleles across rare variants, RVT1 and RVT2, and record *P*-values.
5. Select 15 variants, at random, with probability 4*q_j_*(1 − *q_j_*), as present on the GWA chip. Record the genotypes of each individual in the analysis cohort at all variants on the chip, and apply our two proposed tests of quantitative phenotype association with accumulations of minor alleles across low-frequency variants, recording *P*-values. Apply a standard trend test of quantitative trait association with each SNP on the GWA chip, and record the minimum Sidak-corrected *P*-value to account for multiple comparisons. Estimate population haplotype frequencies across all common SNPs using an expectation maximisation algorithm [Excoffier and Slatkin, 1995]. Apply a haplotype trend test of association with the quantitative trait [Zaykin et al., 2002] across all SNPs on the GWA chip, pooling all rare haplotypes (MAF <1%) in a single class, and record the resulting *P*-value.

For each model, we recorded the proportion of replicates of data for which the *P*-value of each test was less than a 5% significance threshold.
