# Supplementary material for: An Evaluation of Statistical Approaches to Rare Variant Analysis in Genetic Association Studies
Source: Genet Epidemiol. 2009 Oct 6;34(2):188–93. doi: 10.1002/gepi.20450 (PMC2962811; doi:10.1002/gepi.20450)

Supplementary Figure 1. Power of six tests of rare variant association with a quantitative trait at as a function of the percentage of phenotypic variation explained by causal variants in a 50kb region, assuming the trait mean is determined by the presence or absence of minor alleles at any of the causal variants. Results for two models are presented, parameterised in terms of: (i) the maximum MAF of any individual causal variant; and (ii) the total MAF of all causal variants. Power is estimated at a 5% significance level over 10,000 replicates of data. Re-sequencing RVT1: test of phenotype association with the proportion of rare variants, discovered through re-sequencing, at which individuals carry minor alleles. Re-sequencing RVT2: test of phenotype association with the presence/absence of minor alleles in individuals at any rare variant discovered through re-sequencing. GWA <5% RVT1: test of phenotype association with the proportion of low frequency variants on the GWA chip at which individuals carry minor alleles. GWA <5% RVT2: test of phenotype association with the presence/absence of minor alleles at any low frequency variant on the GWA chip. GWA single SNP: standard trend test of quantitative trait association with each SNP on the GWA chip, with Bonferroni correction for multiple testing. GWA SNP haplotypes: haplotype trend test of association with the quantitative trait across all SNPs on the GWA chip.

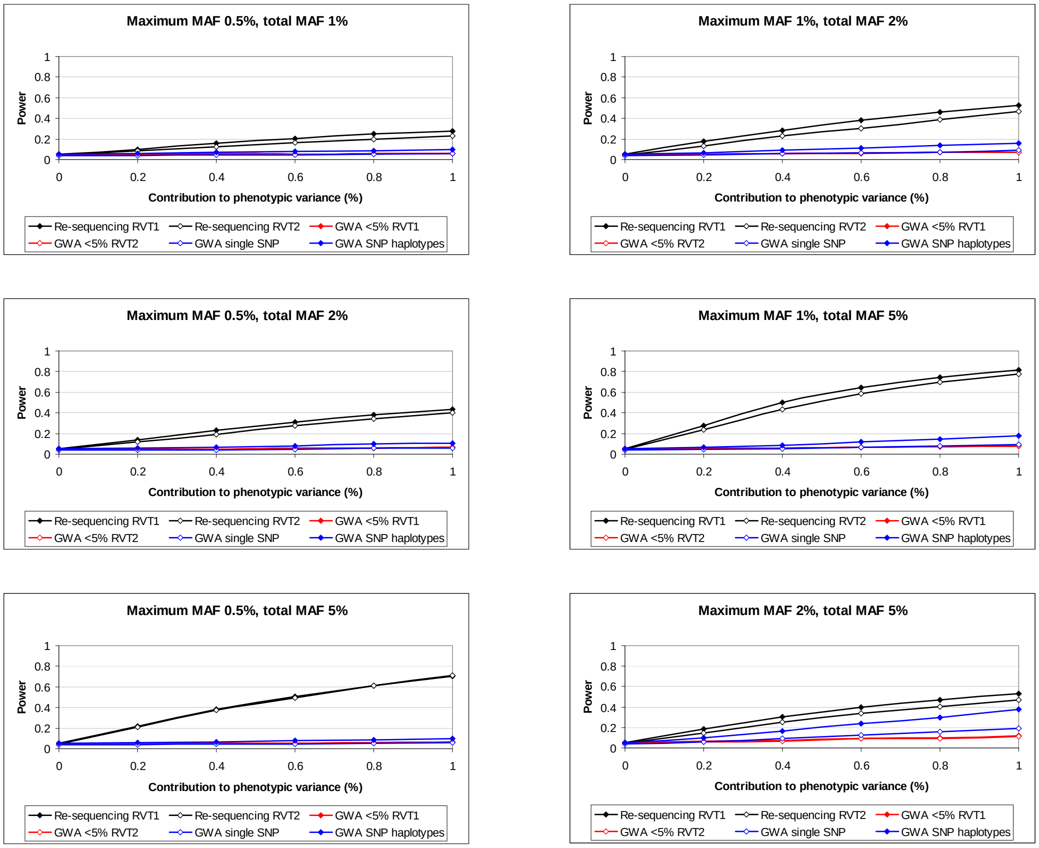

Supplementary Figure 2. Power of six tests of rare variant association with a quantitative trait at as a function of the percentage of phenotypic variation explained by causal variants in a 50kb region, assuming the trait mean is determined by the proportion of causal variants at which minor alleles are present. Results for two models are presented, parameterised in terms of: (i) the maximum MAF of any individual causal variant; and (ii) the total MAF of all causal variants. Power is estimated at a 5% significance level over 10,000 replicates of data. Re-sequencing RVT1: test of phenotype association with the proportion of rare variants, discovered through re-sequencing, at which individuals carry minor alleles. Re-sequencing RVT2: test of phenotype association with the presence/absence of minor alleles in individuals at any rare variant discovered through re-sequencing. GWA <5% RVT1: test of phenotype association with the proportion of low frequency variants on the GWA chip at which individuals carry minor alleles. GWA <5% RVT2: test of phenotype association with the presence/absence of minor alleles at any low frequency variant on the GWA chip. GWA single SNP: standard trend test of quantitative trait association with each SNP on the GWA chip, with Bonferroni correction for multiple testing. GWA SNP haplotypes: haplotype trend test of association with the quantitative trait across all SNPs on the GWA chip.

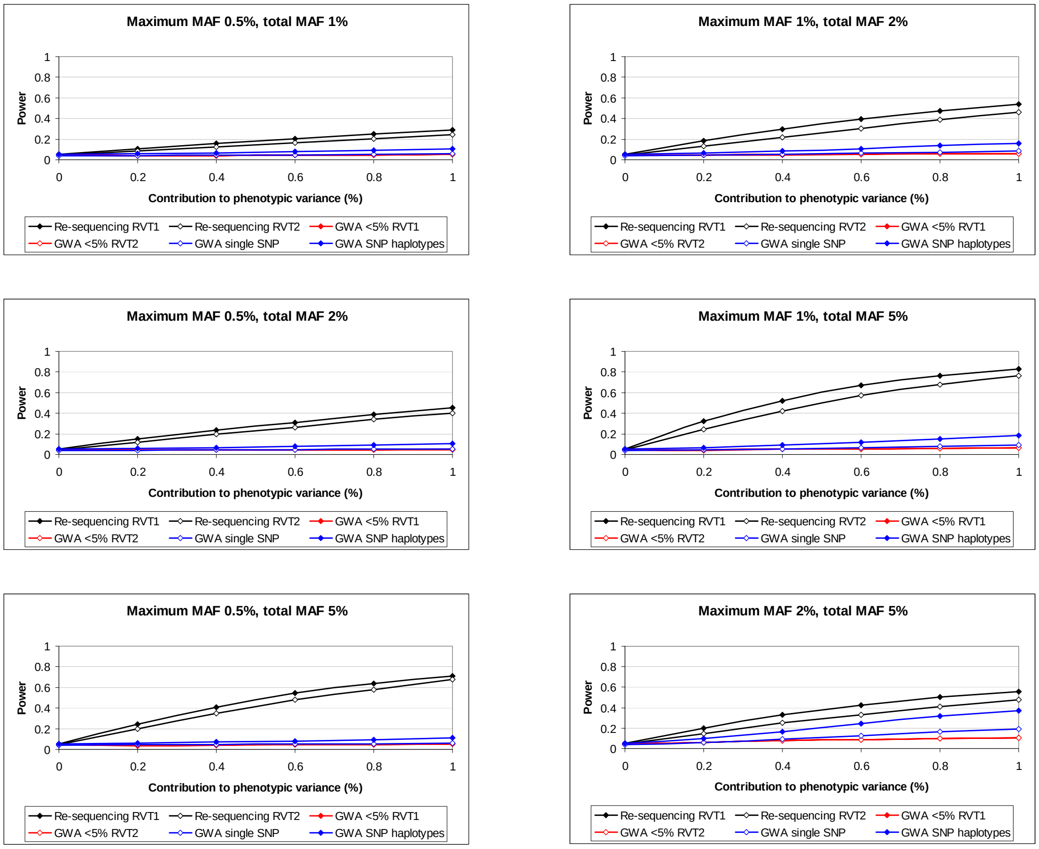

Supplement: Supplementary file 1 [file gepi0034-0188-SD1.pdf]
